# Robotic environmental DNA bio-surveillance of freshwater health

**DOI:** 10.1038/s41598-020-71304-3

**Published:** 2020-09-01

**Authors:** Adam J. Sepulveda, James M. Birch, Elliott P. Barnhart, Christopher M. Merkes, Kevan M. Yamahara, Roman Marin, Stacy M. Kinsey, Peter R. Wright, Christian Schmidt

**Affiliations:** 1 U.S. Geological Survey, Northern Rocky Mountain Science Center, 2327 University Way Suite 2, Bozeman, MT 59715 USA; 2 Monterey Bay Aquarium Research Institute, Moss Landing, CA USA; 3 U.S. Geological Survey, 3162 Bozeman Ave, Helena, MT USA; 4 U.S. Geological Survey, Upper Midwest Environmental Science Center, La Crosse, WI USA; 5 U.S. Geological Survey, Idaho Water Science Center, Boise, ID USA

**Keywords:** Conservation biology, Freshwater ecology, Biotechnology, Biological techniques, Sensors and probes

## Abstract

Autonomous water sampling technologies may help to overcome the human resource challenges of monitoring biological threats to rivers over long time periods and across large geographic areas. The Monterey Bay Aquarium Research Institute has pioneered a robotic Environmental Sample Processor (ESP) that overcomes some of the constraints associated with traditional sampling since it can automate water sample filtration and preservation of the captured material. The ESP was originally developed for marine environment applications. Here we evaluated whether the ESP can provide reliable, timely information on environmental (e)DNA detections of human and fish pathogens and introduced fishes at U.S. Geological Survey streamgage sites in freshwater rivers. We compared eDNA collected via ESP at high frequency (e.g., every 3 h) with manual eDNA collections collected at lower frequency (e.g., weekly). We found that water samples filtered and preserved by ESPs successfully detected the DNA of human pathogens, fish pathogens and introduced fishes. Both ESP and manually collected samples provided similar information about target DNA presence. We suggest that the greatest current benefit of the ESP is the cost savings of high frequency, bio-surveillance at remote or hard to access sites. The full potential of robotic technologies like the ESP will be realized when they can more easily execute in situ analyses of water samples and rapidly transmit results to decision-makers.

## Introduction

Invasive species have serious negative effects on regional and national economies^1,2^. Thus, early detection is a central pillar of most monitoring programs because the earlier an invader is detected, the more likely control efforts will be effective in limiting invader spread and the resulting economic and environmental damages^3^. Environmental DNA (eDNA) sampling has recently emerged as a sensitive, early detection tool because it can detect as little as a single cell from an invasive species by identifying the cellular or extracellular DNA that organisms release into the environment^4^. Despite the ubiquity of eDNA in the aquatic environment, eDNA of targeted taxa is not always well mixed, so high detection probabilities often require intense sampling^5,6^. Therefore, reliable detection requires trained individuals to manually collect water samples over long time periods or across large geographic areas^7^.

**Figure 1:**
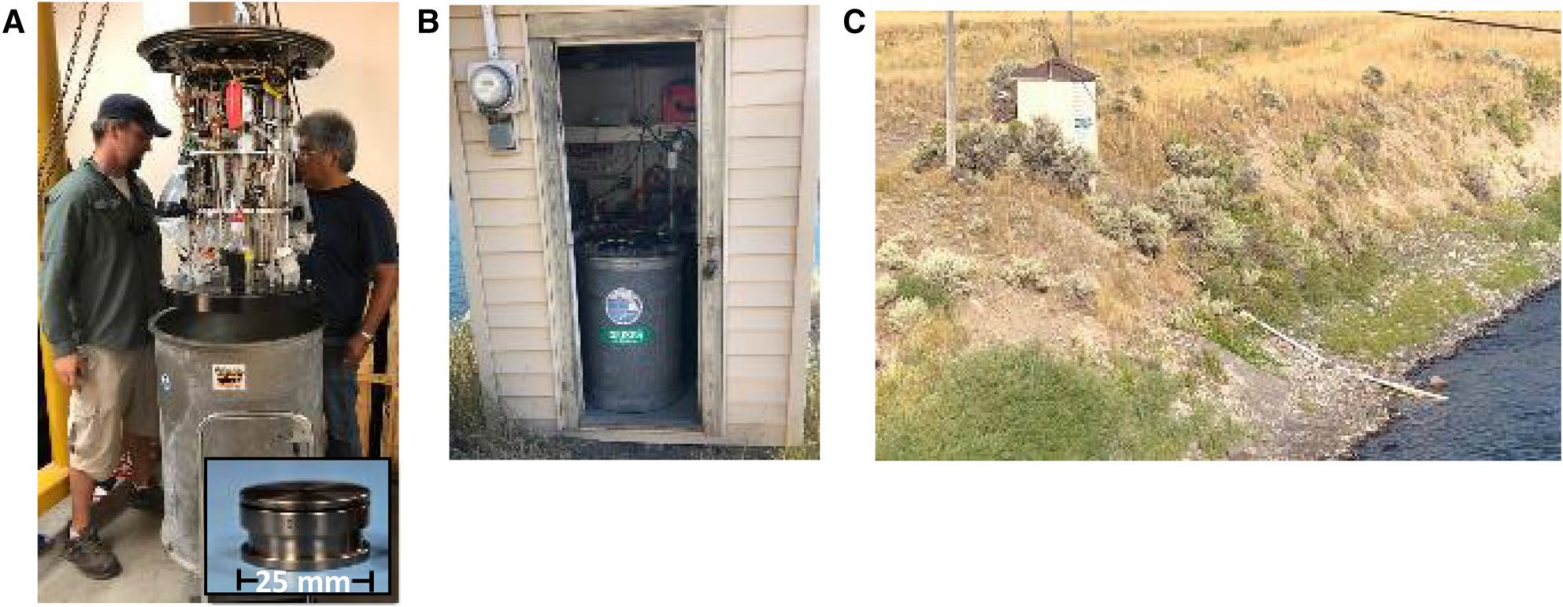
The ESP is an electro-mechanical robot that can autonomously filter and preserve samples. (**A**) About the size of a 50-gal barrel, the ESP carries 132 ‘pucks’ (inset), each designed to hold 25 mm filters. (**B**) The ESP installed in a USGS streamgage station. (**C**) Streamgage station showing tubing run (white pipe) that contained pump and tubing to deliver stream water to the ESP. The ESP communicated via cell phone, and was powered during the deployment via either line power or portable solar arrays. Photo credits: U.S. Geological Survey.

Autonomous, robotic water sampling technologies present an opportunity to overcome the temporal and human resource demands associated with eDNA sampling. Autonomous robots placed within the environment can conduct high frequency sampling, regardless of location, weather or the availability of human resources. The Monterey Bay Aquarium Research Institute (MBARI) has pioneered a robotic instrument called the Environmental Sample Processor (ESP) that overcomes the constraints of regular travel and work schedules, safety concerns with high water flows, and adverse weather. The ESP is a robotic device that can be programmed to automate water sample filtration and preservation of the captured material, or homogenize it for immediate analyses in situ (Fig. 1;^8^).

Various iterations of the instrument have been realized over the past 25 years; here we utilized the “second-generation” (2G) ESP and its archival capabilities to filter water samples and preserve the collected material for later analysis in the laboratory. Real-time, two-way communication via cellular, satellite or radio allow for querying instrument status and augmentation of sampling times or frequency. Although the ESP was originally developed for applications in the marine environment, the ability to operate unattended, over months at a time, made it an attractive option to explore whether the ESP could be helpful to the U.S. Geological Survey (USGS) in carrying out its mission to provide reliable, timely information about the United States’ water resources and aquatic health^9^. Here we report the use of the ESP in the field and at USGS streamgage sites to collect eDNA on sample filters and preserve the filters in situ, with analysis taking place after the filters were returned from the field.

With any new technology, one must show its equivalence or purported advantages over the current methods. This study looks at several species of interest and compares eDNA collected via robot with traditional manual collections to determine if a.) both methods capture similar information about target DNA presence/absence, and b.) higher frequency sampling provides more or better actionable information. Our eDNA targets were human and fish pathogens and introduced fish in the Upper Yellowstone River watershed (Montana, USA) and Upper Snake River (Idaho, USA).

## Materials and methods

### ESP sample processing

The ESP operated autonomously, needing only power, communications and fluid connections through its waterproof pressure housing (Fig. 1). Prior to sample initiation, the ESP was purged completely with nitrogen to reduce oxidative reactions (i.e., DNA degradation) from occurring. At the initiation of sampling, a puck (Fig. 1A cutout) loaded with filter material was placed within a clamp. Valves open to the outside allowed a syringe to sequentially pull water through the puck. Once the target volume was filtered, or the filter was loaded with biomass (i.e., ‘clogged’), filtering stopped and excess water was cleared. Five mL’s of RNAlater preservative was then added to the puck, soaking the filter for 10 min before the excess was evacuated and the puck was returned to storage. Preserved pucks were stored at the ESP temperature, which were similar to ambient air temperatures. The upper limit on the amount of time that an ESP device can operate in the field before DNA quality on a puck is comprised is not known but is at least 21 days^10^. A constant humidity kept the pucks moist, allowing for easy filter removal once the instrument was recovered.

To get water to the ESP, we designed an external sampling module from which the ESP drew water^11^. The sampling module was self-draining, and fed by a submersible pump (WSP-12 V-2 M, Waterra USA Inc., Bellingham, W, USA) installed approximately 0.5 to 2 m below the river water line at each deployment site. To reduce possible carry-over contamination, the sampling pumps, tubing and external sampling modules were flushed with river water for 10 min prior to every sample collection. The sampling port of the ESP itself was cleaned with 10% bleach and a 10% tween-20 solution between samples. At the end of each ESP deployment, pucks were manually removed and filters were aseptically recovered into 2.0 mL screw cap centrifuge tubes and stored at − 80 ºC until molecular analyses were performed.

### Field deployments

**Figure 2:**
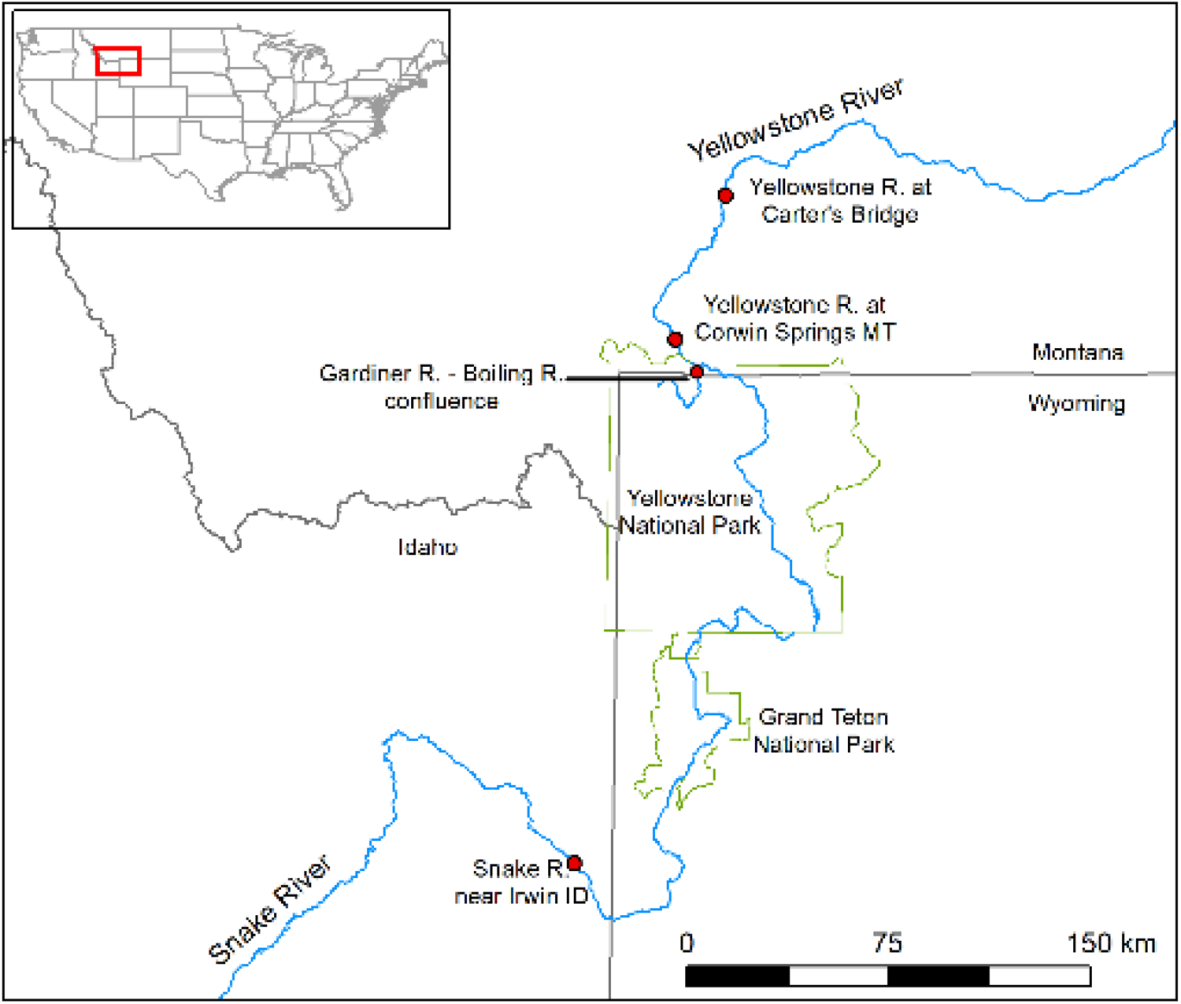
Map of ESP water sampling locations. The inset map shows the location of the Upper Yellowstone River and Upper Snake River in the United States. The larger map shows the sample site locations (filled red circles) on each river relative to Yellowstone National Park and Grand Teton National Park (outlined in green).

We performed initial ESP feasibility studies in Yellowstone National Park (USA; Fig. 2) in September 2017. Here, our goal was to determine if the ESP could be used to sample DNA of the waterborne protozoa, *Naegleria* spp., from a freshwater river where these organisms had previously been detected using standard techniques^12^. We filled 60-L sterilized carboys with water from the confluence of the Boiling and Gardner rivers. Carboys were transported to a lab at Montana State University (Bozeman, Montana) and connected to ESP samplers via tubing and syringe pumps. Water was passed through each filter (5-µm Diapore filters) until the filter became clogged; six samples were filtered.

We then integrated the ESPs into two USGS streamgages on the Yellowstone River in 2018 and one USGS streamgage on the Snake River in 2019, (Fig. 1B,C) where we tested for DNA of the fish pathogen, *Tetracapsuloides bryosalmonae*, the causative agent of salmonid fish Proliferative Kidney Disease (PKD)*.* On the Yellowstone River, we installed ESPs at the streamgage near the upstream and downstream extents of a recent PKD outbreak^13^, USGS 06191500 Yellowstone River at Corwin Springs MT and USGS 06192500 Yellowstone River near Livingston MT, described below as Corwin Springs and Carters Bridge, respectively (Fig. 2). On the Snake River, we installed one ESP at the streamgage 1.5 km downstream of Palisades Reservoir near the upstream extent of a recent PKD outbreak, USGS 13032500 Snake River near Irwin ID. The ESP pucks were loaded with 1.2-µm cellulose nitrate filters. We ran two negative controls (1 L of molecular grade water) through the ESP prior to and at the conclusion of deployment to assess for contamination.

#### Yellowstone River

The ESPs were programmed to collect 1-L samples every 12 h, from Jul 24 to Aug 26 2018, and every 3 h from Aug 27 to Sep 7 2018. The average (± 1 SE) volume filtered per sample was 639 (± 11) mL, indicating that most filters clogged prior to reaching the 1-L target volume. Filter samples were collected at ambient air temperatures ranging from 9.6 to 35.8 °C ($\overline{x}$  = 18.9) at Carter’s Bridge and 8.3–29.0 °C ($\overline{x}$  = 17.1) at Corwin Springs. We compared *T. bryosalmonae* ESP detections to those from manually collected grab samples from shore (6, 250-mL samples per site filtered through 1.2-µm cellulose nitrate filters) collected at weekly frequencies for the entire length of the ESP deployments and at daily frequencies between Aug 27 and Aug 30. Thus, ESP and manual eDNA samples collected at different temporal intervals (3 h, 12 h or weekly) allowed us to evaluate the added value of higher frequency sampling.

We also evaluated the utility of automated high frequency sampling to detect a new invasion by introducing novel DNA of *Scomber japonicas* (mackerel fish) 100 m upstream of each Yellowstone River streamgage. On Aug 27, we introduced 3 kg of canned *S. japonicas* 100 m upstream of the water sampling inlet for each ESP. *S. japonicas* was blended with water, frozen and then placed within metal-wire minnow traps and anchored to the river’s bottom with cement pavers. The ESPs were programmed to sample every 3 h from Aug 27 to Sep 7. Manual grab samples (600 mL) were collected 10 m (n = 3), 100 m (n = 6), and 400 m (n = 3) downstream of the *S. japonicas* in order to test that *S. japonicas* DNA was transported downstream past the water sampling inlet of each ESP. Manual grab samples were collected immediately prior to *S. japonicas* introductions, 3 h post-introduction and then every 24 h for 3 days.

#### Snake River

The ESPs were programmed to collect 2-L samples every 12 h from Jul 17 to Sep 09 and then every 4 h from Sep 10 to Oct 1, 2019. Manually collected grab samples (three, 2-L samples filtered through 1.5-µm glass fiber filters) and negative field controls (1, 2-L sample of deionized water filtered through 1.5-µm glass fiber filters) were collected every 2 weeks following methods in Sepulveda et al.^7^. Filter samples were collected at ambient air temperatures ranging from 3.9 to 30.2 °C ($\overline{x}$  = 20.6). To broaden our taxonomic assessment, we tested these samples for *T. bryosalmonae* DNA, and also for kokanee salmon (*Oncorhynchus nerka*) and dreissenid mussel (*Dreissena* spp.) DNA. *O. nerka* only occur upstream in Palisades Reservoir and at such low abundances that they are not captured by resource managers in annual population surveys^7^. Dreissenid mussels have not yet been observed, but are the principal focus of aquatic invasive species monitoring programs in this region^7^.

### Molecular analyses

Filters were removed from the pucks and then shipped frozen to the USGS Upper Midwest Environmental Science Center (LaCrosse, Wisconsin) for DNA extraction and quantitative PCR analyses. Filters were handled and stored in a dedicated room that is physically separated from rooms where high-quantity DNA extraction and PCR product or high-quality DNA is handled. We used the FastDNA SPIN kit for soil to extract DNA on samples from the Boiling River-Gardiner River confluence, following modifications described in Barnhart et al.^14^. To extract DNA from Yellowstone River and Snake River samples, we used the Investigator Lyse & Spin Basket Kit (Qiagen, Hilden, Germany) in concert with the gMax Mini genomic DNA kit (IBI Scientific), following manufacturer’s instructions, and eluted in 200 µL of buffer. Samples were extracted as site specific batches and one extraction control was collected per batch. We used previously published assays, limits of detection and methods therein for analyses of *Naegleria* spp.^12^, *T. bryosalmonae*^13^, *S. japonicas*^15^, *O. nerka*^7^, and *Dreissena* spp.^16^ (Table 1).

**Table 1:** Primers and probes used in this study.

Target	References	Gene target	Nucleotide sequence 5′–3′	Limit of detection
*Dreissena* spp.	Gingera et al.^16^	16 s	DRE16SF TGGGGCAGTAAGAAGAAAAAAATAA	1 copy
			DRE16SR CATCGAGGTCGCAAACCG	
			DRE16SP FAM/CCGTAGGGATAACAGC/MGBNFQ	
*Naegleria* spp.	Sheehan et al.^12^	ITS	NF GAACCTGCGTAGGGATCATTT	NA
			NR TTTCTTTTCCTCCCCTT ATTA	
*O. nerka*	Sepulveda et al.^7^	COI	SSF CTGCCCTTCTCCTTACGATTT	1 copy
			SSR CAGTGGATCAGAGGAGTGTTAG	
			SSP AATCGCCATCCTGTTCCTCCTGTT	
*S. japonicus*	Sassoubre et al.^15^	COI	324F GCTGAACAGTTTATCCTCCCCTCG	0.1 pg/μL
			430R CCCAAGGATTGAGGAAACACCTGCTAG	
			349P FAM/TGGGAACCTGGCACACGCCGGG/BHQ	
*T. byrosalmonae*	Hutchins et al.^13^	18 s	1337F CGAACGAGACTTCTTCCTT	7 copies
			1426R CTTCCTACGCTTTTAAATAGCG	
			1399P FAM/CCCTTCAATTAGTTGATCTAAACCCCAATT/IBFQ	

We analyzed all samples in four replicate 25 µL reactions containing 2 µL of template DNA, 1 × Perfecta Toughmix (Quantabio), 400 nM forward and reverse primers, and 100 nM probe. Each plate contained 10 no-template PCR controls (one for each sample) using 2 µL of molecular grade water as the template as well as a standard curve with two replicates of 20,000 and 2,000 copy standards and four replicates of 200 and 20 copy standards. The standards were prepared with synthetic gBlocks (Integrated DNA Technologies) containing the amplicon sequences for each assay. Each sample was also analyzed in three replicates with 200 copies of synthetic gBlock spiked in to check for PCR inhibition. Any sample that indicated less than an average of 60 to 70 copies of targeted DNA in these triplicate samples was considered inhibited. Field and extraction negative controls were analyzed as regular samples. No negative controls amplified.

### Analyses

Samples were scored as positive when one or more PCR replicates amplified for the target DNA. We used McNemar’s Exact Test to compare binary qPCR data (detection/non-detection) of *T. bryosalmonae* and *O. nerka* DNA between ESP and manually collected samples in the Yellowstone and Snake rivers.

## Results

### Feasibility study

*Naegleria* spp. DNA was detected in all PCR replicates from the seven ESP samples, thus demonstrating that the ESP could be used to capture target DNA from a freshwater river where these organisms had previously been detected using standard techniques . Further sequencing analysis is needed to confirm species identity (e.g., *N. fowleri*), as the assay is only specific to the *Naegleria* genus.

### USGS streamgages

#### Yellowstone River

**Figure 3:**
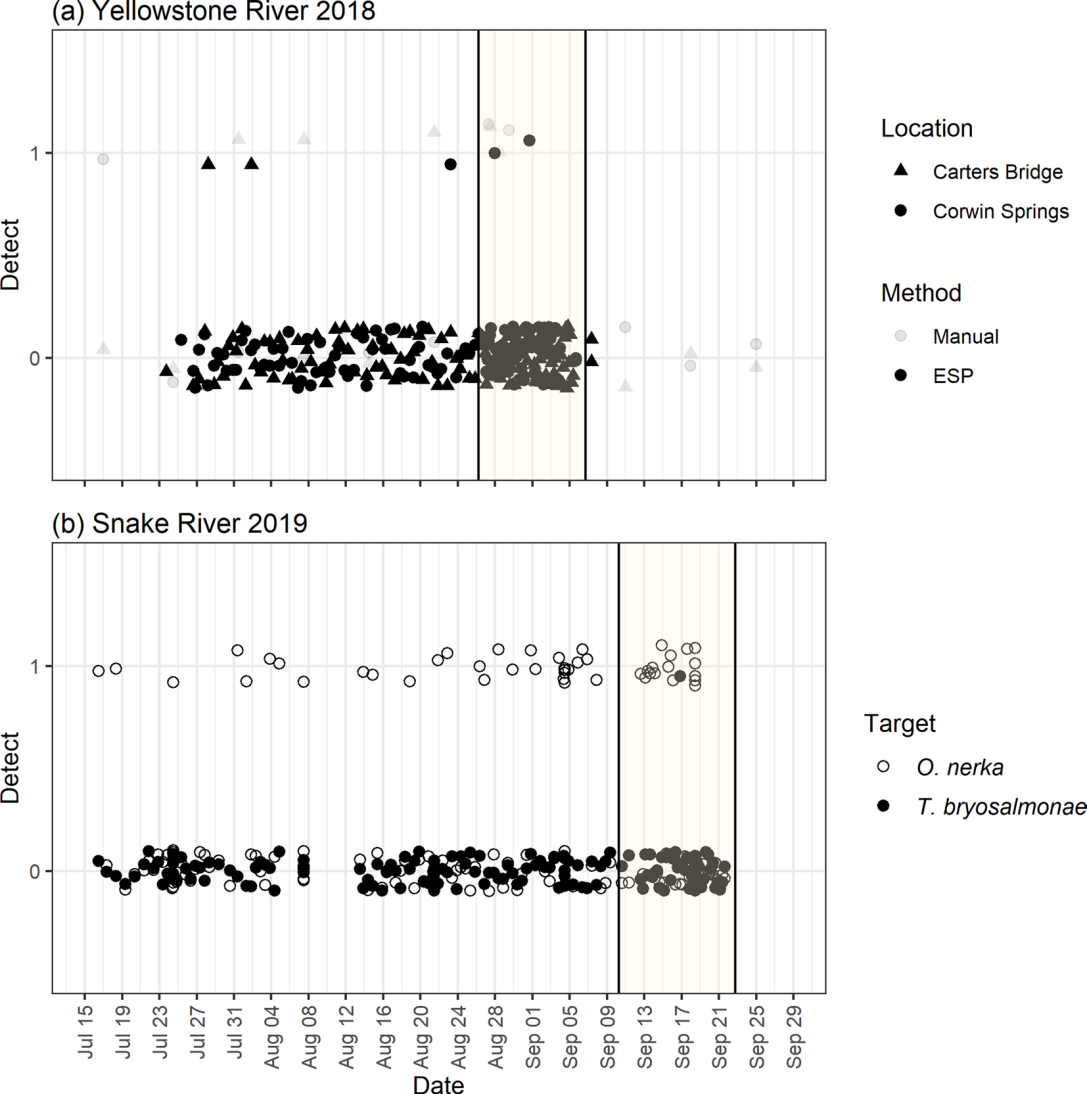
Detection history of ESP and manually-collected grab samples from two locations on the Yellowstone River in 2018 (**a**) and one location on the Snake River in 2019 (**b**). Positive detections were those with at least 1 PCR replicate amplified. For clarity, symbols are jittered around the no detection (0) or detection (1) line. *T. bryosalmonae* DNA was targeted at Yellowstone River locations and *O. nerka* and *T. bryosalmonae* DNA were targeted at the Snake River location. Shaded areas indicate time period when samples were collected every 3 h (**a**) or 4 h (**b**) rather than every 12 h.

**Figure 4:**
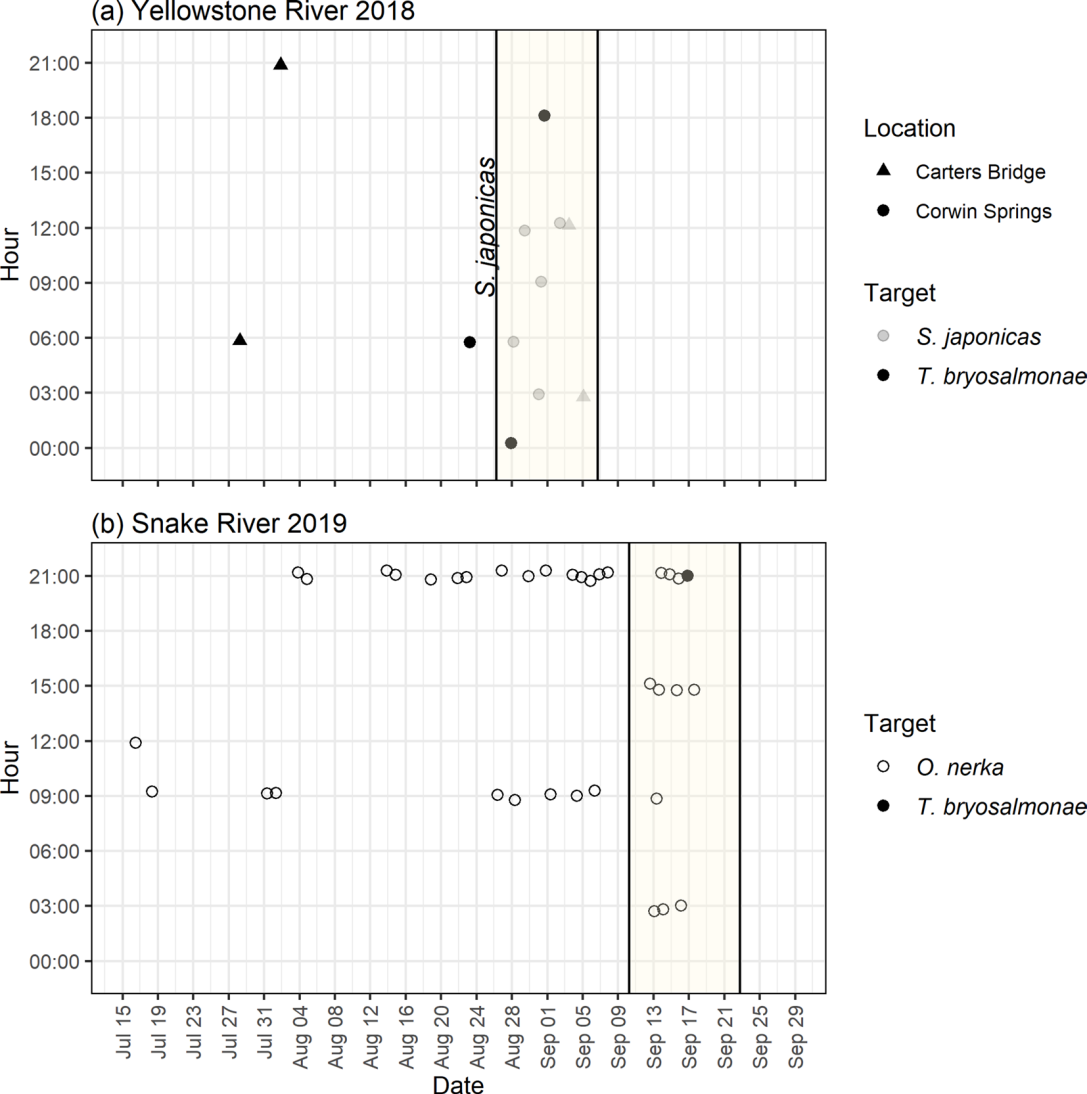
Temporal distribution of positive detections from ESP samples collected from two locations in the Yellowstone River in 2018 (**a**) and one location in the Snake River in 2019 (**b**). *S. japonicas* slurry introductions to Yellowstone River locations are indicated by the vertical line on Aug 27. *S. japonicas* and *T. bryosalmonae* DNA were detected at Yellowstone River locations and *O. nerka* and *T. bryosalmonae* DNA were detect at the Snake River. Shaded areas indicate time period when samples were collected every 3 h (**a**) or 4 h (**b**) rather than every 12 h.

*T. bryosalmonae* DNA was detected in at least one PCR replicate in three of 128 ESP samples at Corwin Springs and in two of 128 ESP samples at Carters Bridge (Fig. 3). Three of these detections occurred during lower frequency sampling (every 12 h), which spanned 4 weeks, and two detections occurred during higher frequency sampling (every 3 h), which spanned 2 weeks (Fig. 4). Interestingly, detections during the higher frequency period only occurred outside of typical workday times (e.g., 08:00–17:00) when manual samples would not normally be collected (Fig. 4).

Over the same time period, *T. bryosalmonae* DNA was detected in six of 55 manually-collected samples (Fig. 3). All but one of these detections occurred within a day of an ESP detection. No difference was observed between the detection/non-detection of *T. bryosalmonae* when comparing the ESP to manual sampling methods (χ2 = 6, *p* = 0.13, n = 53).

*S. japonicas* DNA was detected in ESP samples at both locations (Fig. 4). At Carters Bridge, *S. japonicas* DNA was detected in ESP samples ~ 1 week after *S. japonicas* introduction; *S. japonicas* DNA was not detected in any grab samples from the same location. At the other sampling distances, *S. japonicas* DNA was only detected in grab samples from 10 m downstream at 3 days. At Corwin Springs, *S. japonicas* DNA was first detected in ESP samples ~ 1.5 days after *S. japonicas* introduction and then again at 3, 5 and 7 days. *S. japonicas* DNA was detected in grab samples from this location at 1, 2 and 3 days. *S. japonicas* DNA was also detected in grab samples at 10 m and 400 m after only 3 h, at 400 m after 1 day, and at 10 m after 2 days. No difference was observed between the detection/non-detection of *S. japonicas* DNA when comparing the ESP to manual sampling methods (χ2 = 6, *p* = 0.29, n = 26).

#### Snake River

The ESP collected, filtered and preserved 128 field samples and technicians collected 30 manual samples. We detected *T. bryosalmonae* DNA in only one ESP sample and in no manual samples (Fig. 3). We did not detect dreissenid mussel DNA using either sampling approach. We detected *O. nerka* DNA in 35 ESP samples and 11 manual samples, though no difference was observed between the detection/non-detection of *O. nerka* DNA when comparing the ESP to manual sampling methods (χ2 = 1.69, *p* = 0.19, n = 59). *O. nerka* DNA detections from ESP samples were spread out across dates, while manual detections were clumped and often occurred on the same day (Fig. 3). In fact, manual samples failed to detect *O. nerka* DNA for most of August whereas the ESP had 13 detections. Higher frequency sampling (every 6 h) resulted in seven additional detections than lower frequency sampling (every 12 h; Fig. 4).

## Discussion

The Upper Yellowstone and Snake rivers share similar concerns with regard to invasive species and disease-causing organisms, as both are premier river fisheries stimulating over USD$150 million to the local economy and serve as headwaters for a substantial portion of the United States^17,18^. Thus, surveillance of river health is a paramount concern. Surveillance approaches that provide early detection warnings for a broad range of taxa are especially needed since the list of potential introduced species is unbounded. Additionally, the extensive area to be monitored for invasive species makes it difficult to send technicians to all areas of concern. Technology may provide a solution in the form of autonomous sampling robots, capable of collecting and preserving samples, or ultimately processing those samples in real time.

We found that water samples filtered and preserved by ESPs could be used to detect the DNA of waterborne protozoa, *T. bryosalmonae* parasites, introduced *O. nerka* and even novel DNA added to the river from canned *S. japonicas*. In this study, both ESP and manually collected samples provided similar information about target DNA presence (Fig. 3). For instance, the ESP detected *O. nerka* DNA in 28% (35 of 128) of Snake River samples, compared to manual methods that detected *O. nerka* DNA in 33% (10 of 30) of samples. These similar detection rates are encouraging. However, the high frequency sampling enabled by the ESPs generated much more data so provided stronger weight of evidence for inferring target species presence and absence. For example, a negative ESP result provided some confidence that the target species DNA was absent, while a ‘no data’ result from less frequent manual sampling provides no information. If resources are available to support frequent sampling, then either method would provide similar results, especially when integrated into a probabilistic framework for modeling uncertainty in the detection of the target species DNA^19^.

The initial costs of the ESP, and robotic technology in general, are still large relative to human labor. But when the ‘incidental’ costs required for high frequency, manual sample collection are factored, the equation begins to change. For manual grab samples, collection of three field samples and one field negative control at a USGS streamgage requires an additional 2 h per site per visit if the sampling is part of routine USGS hydro-technician monitoring activity^7^. Travel time (personnel time, per diem, gas, etc.) becomes an additional expense if eDNA surveillance is needed at a frequency greater than the 4–6 week routine monitoring schedule. Travel costs required for high frequency manual monitoring can quickly escalate in expansive areas like the western United States. High frequency surveillance by hydro-technicians would be demanding and costly to repeatedly sample a site throughout an entire season and would limit the number of sites that they could sample. Thus, we suggest that a major benefit of the ESP is the cost savings of eDNA surveillance at remote or hard to access sites.

While high-frequency sampling may better capture unique or rare events, it may not be advantageous in all situations. The two years we sampled for *T. bryosalmonae* on the Yellowstone and Snake Rivers were marked by extremely low abundances of this fish parasite. We detected *T. bryosalmonae* DNA in both manual and ESP sampled water, but the higher sampling frequency of the ESP (e.g., 2–8 times per day vs. weekly or biweekly) did not improve parasite DNA detection rates. This may be explained by the rarity of the parasite these two years.

The USGS streamgage network would be ideal to test the scalability of this robotic technology. With > 8,200 streamgages nationwide, the USGS has an existing infrastructure where ESP-collected biological data could be paired with existing physio-chemical data. Ultimately, this should improve near-real time modeling and forecasting of when introduced species and pathogens are likely to become invasive or cause disease. Currently, ESP freshwater technology is still in the proof-of-concept phase and is too cumbersome and expensive (e.g., a 2G ESP costs > USD$100,000) for a complete roll-out to the nation’s streamgage network.

Finally, collecting samples for later return and analysis simply pushes the work and expense to a laboratory, which will be inundated with samples to process, and then only provide a look at the past, describing what organisms were detected in the past month(s). In the version of the ESP that we evaluated, there was a lengthy time lag between when a sample was collected and when it was analyzed, since the ESP must complete its entire sampling mission before filters are removed and sent to a molecular lab for analysis. The full potential of robotic technologies like the ESP will be realized when they can execute in situ analyses of water samples and transmit results to decision-makers. This near real-time analysis has been demonstrated in marine environments using in situ qPCR^11,20,21^, but these in situ modules are still in research and development phases. Future directions should include evaluation of this qPCR module in freshwater and development of expedient data processing software that integrates eDNA results and physio-chemical covariates collected from the USGS streamgage network. When in situ analysis becomes commonplace in environmental contexts, we will then realize the ultimate goal of timely, up-to-date information that can be used to minimize negative outcomes and improve management decision making.

## Data Availability

Data are available at https://www.sciencebase.gov/catalog/item/5e41ab1ce4b0edb47be63b4a
